# Head of bed elevation to relieve gastroesophageal reflux symptoms: a systematic review

**DOI:** 10.1186/s12875-021-01369-0

**Published:** 2021-01-19

**Authors:** Loai Albarqouni, Ray Moynihan, Justin Clark, Anna Mae Scott, Anne Duggan, Chris Del Mar

**Affiliations:** 1 Institute for Evidence-Based Healthcare, Faculty of Health Sciences and Medicine, Bond University, 14 University Dr, Robina, QLD 4229 Australia; 2 Australian Commission on Safety and Quality in Health Care, Sydney, NSW Australia

**Keywords:** GORD, Non-pharmacological interventions, Proton pump inhibitors, General practice

## Abstract

**Background:**

Overuse of proton pump inhibitors (PPIs) – frequently used for relieving symptoms of gastroesophageal reflux disease (GORD) – raises long-term safety concerns, warranting evidence-based non-drug interventions. We conducted a systematic review to evaluate the effect of head-of-bed elevation on relieving symptoms of GORD in adults.

**Methods:**

We included controlled trials comparing the effect of head-of-bed elevation interventions to control in adults with GORD. Two independent reviewers screened articles, extracted data, and assessed quality of included studies. Primary outcomes were changes in GORD symptoms and use of PPIs.

**Results:**

We screened 1206 records; and included five trials (four cross-over and one factorial) comprising 228 patients. All five included trials were judged to be at high-risk of performance bias and four of selection bias. Of five included trials, two used ‘bed blocks’ under the bed legs; one used ‘sleeping on a wedge’ pillow, and two used both. High heterogeneity in outcome measures and reported outcomes data precluded meta-analyses. The four studies that reported on GORD symptoms found an improvement among participants in the head-of-bed elevation; a high-quality crossover trial showed a clinical important reduction in symptom scores at 6 weeks (risk ratio of 2.1; 95% CI 1.2 to 3.6). These results are supported by the observed improvement in physiological intra-oesophageal pH measurements.

**Conclusions:**

Methodological and reporting limitations in available literature preclude definitive recommendations. However, head-of-bed elevation could be still considered as a cheap and safe alternative to drug interventions with unfavourable safety profiles.

**Protocol registration:**

Open Science Framework: http://osf.io/2hz3j

**Supplementary Information:**

The online version contains supplementary material available at 10.1186/s12875-021-01369-0.

## Background

Proton pump inhibitors (PPIs) are among the most commonly prescribed medications worldwide [1]. PPIs are usually prescribed to treat common upper gastrointestinal symptoms often diagnosed as Gastroesophageal Reflux Disease, GORD (i.e. also known as GERD or reflux disease) [2]. While PPIs are effective at controlling these symptoms, there is evidence of great variation in prescribing rates [3], and widespread overuse (e.g. with estimates that between one-quarter and two-thirds of patients may be taking them inappropriately) [1, 4–6]. Long term use of PPIs has been linked to a potential increased risk of fractures [7, 8]; pneumonia [9]; and *Clostridium difficile* infection [10]. Many deprescribing initiatives are underway to reduce the use of PPIs [1, 11]. Against this backdrop of concern about widespread overuse, patient harm and waste, it is timely to investigate the evidence supporting non-drug interventions for the symptoms of GORD [12, 13].

Gastrointestinal symptoms such as heartburn, dyspepsia, and regurgitation are common - one-in-five adults report one of these symptoms at some point in their lives [14]; and they are a very common reason for primary care consultations [15]. Although these symptoms are highly prevalent and can be mild or transient, adults experiencing these symptoms are often diagnosed with GORD. Guidelines recommend a stepwise approach for managing GORD symptoms, beginning with non-drug interventions including lifestyle modifications (e.g. weight loss, smoking cessation, and avoiding late or evening meals), and progressing to drug and surgical interventions when needed [2, 16]. However, the widespread use of drug interventions such as PPIs has rendered non-drug interventions underused and unfashionable, and its full potential as an effective addition or alternative to drug interventions has been under-researched and under-utilised [17].

One promising, easy-to-adopt, non-drug intervention is elevating the head of the bed, which may also be used to avoid, or to lower the dose required of PPIs [12]. A potential mechanism of action is by reducing the oesophageal exposure to stomach acid and increasing the clearance of acid from the oesophagus [18]. Trials have been conducted to evaluate this simple intervention [19, 20], however, we are unaware of any recent high quality systematic review summarizing evidence to inform practice. The aim of this study was to do a systematic review of controlled trials that evaluated the effect of head-of-bed elevation or positioning on relieving GORD symptoms among adults.

## Methods

### Design

This systematic review is reported following the Preferred Reporting Items for Systematic Reviews and Meta-Analyses (PRISMA) statement [21]. and the review protocol was developed prospectively and registered on the Open Science Framework (osf.io/2hz3j).

### Eligibility criteria

#### Participants

We included studies of adults with symptoms suggestive of GORD (of any severity and as diagnosed in each study).

#### Interventions

We included studies evaluating change of the head-of-bed position interventions including (i) head-of-bed elevation (either by sleeping on a wedge pillow – ‘*sleeping on a wedge’* – or raising the legs of the head of the bed by blocks - *‘bed blocks’*); (ii) left lateral sleep position; (iii) or both. Studies evaluating interventions of interest together with co-interventions (e.g. PPIs) were included, as long as the effect of the intervention of interest could be isolated (e.g. head-of-bed elevation plus PPIs vs PPIs alone).

#### Comparators

We included studies where the comparator was control (i.e. no change to head-of-bed elevation e.g. flat position) or right lateral sleep position.

#### Outcomes

Primary outcomes were changes in GORD symptoms and use of PPIs. Secondary outcomes included physiological measurements of intra-oesophageal pH (e.g. acid exposure/reflux episodes), disease progression, and adverse events.

##### Study design

We included randomized and non-randomized controlled trials (RCTs and non-RCTs). We excluded before-after studies with no control group, observational studies, and review articles. We included publications available as abstract only (e.g. conference abstract) only if they reported adequate information required for inclusion.

### Search strategies to identify studies

One of the authors, a senior information specialist (JC), searched PubMed, Embase, Cochrane CENTRAL and CINAHL (from inception until 23 June 2020). The search string was designed for PubMed and translated for use in other databases using the Polyglot Search Translator [22]. The complete search strings for all databases are provided in Appendix 1. We also searched clinicaltrials.gov and World Health Organization’s International Clinical Trials Registry Platform (via the Cochrane Library on 23 June 2020) for any relevant registered ongoing or unpublished trials. We supplemented our database searches with a forwards and backwards citation search of all included studies in Scopus database (on 25 June 2020). No restrictions by language or publication date were imposed.

### Study selection and screening

Two review authors (LA, RM) independently screened the titles and abstracts for inclusion against inclusion criteria; one author (JC) retrieved full texts, and two authors (LA, RM) screened the full texts for inclusion. Any disagreements were resolved by discussion, or referred to a third author (AD, CDM).

### Data extraction and risk of bias assessment

Two review authors (LA, RM) independently extracted data into a prespecified, pilot-tested data extraction form – including the following data on study characteristics; participants; interventions; comparisons; and outcomes.

Two review authors (LA, RM) independently assessed the risk of bias for each included study using the Cochrane Collaboration’s tool for assessing risk of bias [23]. Any disagreements were resolved by discussion or by referring to a third author (AD or CDM).

### Data synthesis

We could not undertake meta-analyses because it was not possible or appropriate (i.e. no sufficient comparable data measuring the same outcome). Therefore, we synthesized the results narratively, reporting the results for each outcome separately – following the Synthesis without meta-analysis (SWiM) in systematic reviews guidance [24]. For continuous outcomes, we used mean difference (with 95% CIs) or standardized mean difference, as appropriate. For dichotomous outcomes, we used risk ratios (with 95% CIs) for results reporting the number of people with an event, as appropriate.

## Results

**Fig. 1:**
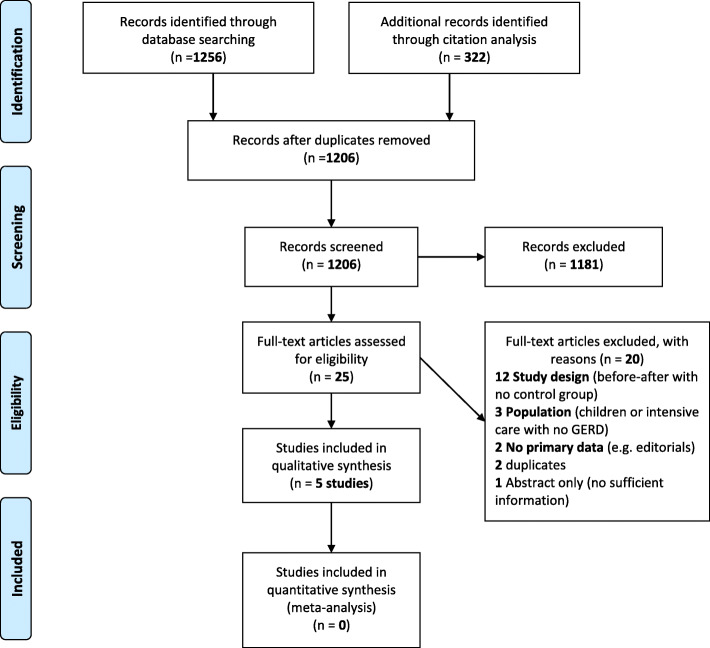
PRISMA flow diagram of included articles

We identified 1256 records through database searching and 356 through forward-backward citation analysis for a total of 1206 records to screen after deduplication. We excluded 1181 records after screening titles and abstracts and obtained 25 records for full-text screening. We excluded 20 full-text articles with reasons for exclusion recorded. (Fig. 1) We also screened 36 clinical trial registries and found one relevant trial that we already identified in database searches. We included five studies in the narrative synthesis.

### Characteristics of included studies

Of all the five included randomised controlled trials, two were conducted in the United Kingdom [25, 26], with the others in the United States [27], Taiwan [28], and Columbia [29] (Table 1). Three studies were published in the 1970s and 1980s [25–27], with the two most recent published in 2019 and 2020 [28, 29]. All studies were small, with total number of participants ranging from 14 to 71, and were of short-term duration, with the longest intervention being for 6 weeks [26, 29]. Four studies were cross-over trials [25, 27–29] and one was a factorial RCT [26]. All trials had participants with gastroesophageal reflux symptoms, with one trial conducted among participants who had previously had oesophageal cancer and undergone esophagectomy and gastric tube reconstruction [28]. All included studies were reported in English except for one reported in Spanish, which was also reported in English in clinicaltrials.gov (NCT02706938) [29].

**Table 1:** Characteristics of included studies (*n* = 5)

Study author, year, country, study design	Participants, condition or symptom, setting, age	Intervention	Comparison	Co-interventions	Outcomes assessed in this review
Morales et al., 2020, Columbia cross-over RCT [29]	**65** participants with GORD-associated sleep disturbance recruited from a hospital outpatient unit with mean age of 56 years	*Bed blocks + PPIs and/or sodium alginate* Head-of-bed elevation for 6 weeks at home using 20 cm wooden blocks under bed	*Lying flat* No Head-of-bed elevation as clinically indicated.	PPIs and/or sodium alginate	Gastroesophageal reflux symptoms Patient preferences Adverse Events
Huang et al., 2019, Taiwan, cross-over RCT [28]	**14** participants with oesophageal cancer and nocturnal reflux symptoms or reflux esophagitis, recruited from hospital database, with mean age of 62 years	*Sleeping on a Wedge + PPIs* Using a 20 cm high wedge-shaped pillow at home (with an elevation angle of 20 degree) for 2 weeks + PPIs	*PPIs only* Not using the pillow for 2 weeks	One pillow of ≤8 cm high	Gastroesophageal reflux symptoms
Hamilton et al., 1988, United States, cross-over RCT [27]	**15** participants with chronic reflux symptoms and endoscopic evidence of erosive esophagitis, recruited from hospital outpatients, aged between 51 and 74 years	*Sleeping on a Wedge* Using a 25 cm high foam wedge (with an elevation angle of 22 degree) for one night. *Bed blocks* Head-of-bed elevated using 20 cm high metal cones under the bed legs for one night.	*Lying flat* One pillow on a standard hospital bed for one night. ^b^	All anti-reflux medications stopped. Other chronic medications allowed.	Intra-oesophageal pH measurement (Acid exposure/reflux episodes/acid clearance time) Patient preferences
Harvey et al., 1987, United Kingdom, factorial RCT^a^ [26]	**71** participants with severe gastro-oesophageal reflux, recruited from a hospital, with a median age of 59 years	*Bed blocks* Head-of-bed raised on 20 cm blocks (with 10% elevation slope) for 6 weeks at home.	*Lying flat* No bed elevation	Antacid tablets as needed	Gastroesophageal reflux symptoms Adverse Events
Stanciu et al., 1977, United Kingdom, cross-over trial [25]	**63** participants with typical symptoms of Gastroesophageal reflux, recruited within a hospital, with mean age of 49 years	*Bed blocks* Head-of-bed elevated with 28 cm blocks during part of the night *Sleeping on a Wedge* Sitting propped up during part of the night	*Lying flat* One or two pillows during part of the night	None	Gastroesophageal reflux symptoms Intra-oesophageal pH measurement (Acid exposure/reflux episodes/acid clearance time)

^a^Factorial RCT – 2 factors ranitidine and head-of-bed elevation. ^b^All groups in hospital and for each group a pillow was allowed

In terms of intervention details (i.e. how elevation was achieved), two trials used *‘bed blocks’* (i.e. 20 cm blocks under the legs of the head of the bed) [26, 29], one trial used *‘sleeping on a wedge’* (i.e. sleeping on a 20 cm wedge-shaped pillow) [28], and two trials used both interventions i.e. 20-28 cm *‘bed blocks’* and *‘sleeping on a wedge’* (as two separate intervention arms) [25, 27]. None of the included studies evaluated the left lateral sleep position as an intervention.

### Risk of bias assessment

**Fig. 2:**
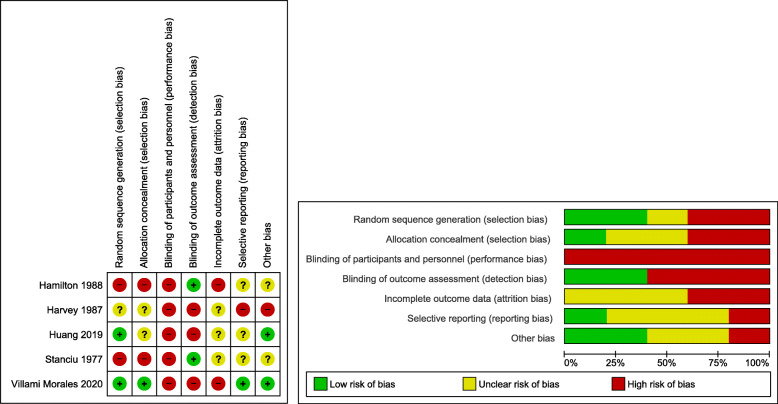
Risk of bias assessment in included studies both in individual studies and domains (left) and in summary (right)

All five included studies were judged to be at high risk in two or more of the domains of risk of bias. All five studies were judged to be at high risk of performance bias (either blinding of patients and personnel or outcome assessors), and four at high or unclear risk for selection bias (either random sequence generation or allocation concealment) (Fig. 2).

### Main findings

Heterogeneity, especially of outcome measures and the reporting of outcome data, precluded meta-analyses of the primary and secondary outcomes. Therefore, we reported the results narratively grouped for each outcome (Table 2).

**Table 2:** Main results reported in included studies (*n* = 5)

Studies	Patient-reported gastroesophageal reflux symptoms	Intra-oesophageal pH measurement	Preferences	Adverse events
Morales, 2020, Columbia cross-over RCT [29]	**Outcome measures (validated)** Reflux Disease Questionnaire Score, a 6-point score, with a higher score meaning a worse outcome and a change of ≥0.6 points considered clinically meaningful **Findings (improved)** Mean difference (MD) in change scores at 6 weeks of 1.327, 95% CI 0.626 to 2.027. 27 of 39 [69.2%] participants in the head-of-bed elevation group were more likely to report a clinically meaningful change of ≥10% in their symptom scores at 6 weeks compared to 13 [33.3%] in the control group. Risk ratio (RR) of 2.08; 95% CI 1.19 to 3.61).	**NR**	63.2% (95% CI 50.6 to 75.7%) of the 57 participants preferred the head of bed elevation to control.	39 of 63 participants in head-of-bed elevation group reported adverse events at 14 weeks compared to none on the control group (of 54): 20 bed slipping; 4 bed unsteadiness; 10 varicose vain pain; 7 musculoskeletal pain; and 1 sexual activity interference and headache each.
Huang, 2019, Taiwan, cross-over RCT [28]	**Outcome measures (validated)** Modified Dysfunction after Upper Gastrointestinal Surgery, a 30-point score, with higher scores means better outcomes. **Findings (improved)** MD in change scores at 2 weeks − 5.1, 95% CI − 7.6 to − 2.6	**NR**	**NR**	**NR**
Hamilton, 1988, United States, cross-over RCT [27]	**NR**	**Outcome measures** *Acid exposure*, defined as percent of total time intra-oesophageal pH remained ≤4; *Reflux episodes,* defined as drop of intra-oesophageal pH to < 4; *Acid clearance time*, defined as the total time that intra-oesophageal pH < 4 by the number of reflux episodes. **Findings (improved)** A reduction in acid exposure in both sleeping on a wedge (mean% 14.8, 95% CI 12.2 to 17.5 vs control: 21.2, 95% CI 18.4 to 23.7) and head-of-bed elevation groups (mean% 15.3, 95% CI 11.7 to 18.8 vs control: 21.2, 95% CI 18.4 to 23.7) compared to the control. A non-statistically significant reduction in the number of reflux episodes and acid clearance time in any of the two groups compared to the control.	60% (9 of 15) preferred head-of-bed elevation, 33.3% (5 of 15) preferred sleeping on wedge, and 6.7% (1 of 15) preferred neither.	**NR**
Harvey, 1987, United Kingdom, factorial RCT [26]	**Outcome measures** An overall improvement in symptoms and a 4-point-sclae of gastroesophageal reflux, retrosternal pain, epigastric pain, and dysphagia. **Findings (improved)** 23 of 32 [71.9%] participants in the head-of-bed elevation group are more likely to report an overall improvement in symptoms at 6 weeks compared to 17 of 31 [54.8%] - unadjusted OR 2.1, 95% CI 0.74 to 5.99; adjusted OR 3.1, X^2^ = 4, *p* < 0.005. A statistically significant reduction in symptom scores of gastroesophageal reflux and retrosternal pain but not epigastric pain and dysphagia.	**NR**	**NR**	2 of 32 participants in the head-of-bed elevation group reported adverse events both resolved and neither discontinued: 1 bed slipping and 1 sexual activity interference.
Stanciu, 1977, United Kingdom, cross-over trial [25]	**Outcome measures** Number of reflux symptoms (i.e. heartburn or acid regurgitation) **Findings (improved)** Compared to control group, a statistically significant reduction in the number of reflux symptoms during the intervention (6 h) in the head-of-bed elevation group (2.0 ± 1.2 vs. 3.0 ± 2.4) and sleeping on a wedge groups (2.4 ± 1.4 vs. 3.4 ± 2.2).	**Outcome measures** *Acid exposure*, defined as percent of total time that intra-esophageal pH remained < 4; *Reflux episodes,* defined as a drop in intra-esophageal pH by 2; *Acid clearance time*, defined as the total duration of reflux by the number of reflux episodes. **Findings (improved)** A statistically significant reductions in acid exposure and reflux episodes in the head-of-bed elevation group (acid exposure: 6.7 ± 7.6; reflux episodes per participant: 3.7 ± 1.9) compared to control group (acid exposure: 14.0 ± 15.3; reflux episodes per participant: 6.2 ± 3.9) but no significant difference in sleeping on a wedge group compared to control group in terms of acid exposure (7.7 ± 11.7 vs 8.9 ± 9.7) and reflux episodes (5.4 ± 3.8 vs. 4.8 ± 3.2).	**NR**	**NR**

*Abbreviations*: *NR* Not reported, *RCT* Randomized controlled trial, *CI* Confidence interval, *RR* Risk ratio, *OR* Odds ratio

#### Gastroesophageal reflux symptoms

All the four (of the five included) studies that we identified evaluating the impact of head of bed elevation on patient-reported gastroesophageal reflux symptoms found an improvement among participants in the head-of-bed elevation intervention arm [25, 26, 28, 29].

A crossover randomized trial analysed the change in GORD symptoms among 39 participants (out of the 65 enrolled participants) at 6 weeks using a 6-point scale, the Reflux Disease Questionnaire Score, with a higher score meaning a worse outcome and a change of ≥0.6 points considered clinically meaningful [29]. The trial found that participants in the head-of-bed elevation group were more likely to report a clinically meaningful change of ≥10% in their symptom scores at 6 weeks compared to the participants in the control group (27 [69%] versus 13 [33%], risk ratio of 2.1; 95% CI 1.2 to 3.6); with a mean difference in change scores of − 1.3, 95% CI − 2.0 to − 0.6).

A crossover trial of 63 participants [25], evaluating the effect of head-of-bed elevation using *‘bed blocks’* or *‘sleeping on a wedge’*, found a reduction in the number of reflux symptoms (i.e. heartburn or acid regurgitation) reported during the intervention (i.e. half-night) in the head-of-bed elevation arm (mean difference in *‘bed blocks’* -1, 95% CI − 2.2 to − 0.1; and ‘*sleeping on a wedge’* -1, 95% CI − 1.9 to − 0.01) compared to the control group.

A factorial RCT [26], evaluating the effect of ranitidine and head-of-bed elevation among 71 participants, found that participants in the head-of-bed elevation group were more likely to report an overall improvement in their symptoms at 6 weeks compared to the control group (72% [23 of 32] vs. 55% [17 of 31]; unadjusted odds ratio 2.1, 95% CI 0.7 to 6.0; odds ratio adjusted for ranitidine use 3.1, *p* < 0.005). It also found a reduction in the reported scores, measured on a 4-point-scale, of gastroesophageal reflux and retrosternal pain symptom in the head-of-bed elevation group compared to the control group. There was no statistically significant difference in overall improvement of symptoms reported by adults receiving the head-of-bed elevation only compared to adults receiving ranitidine only (59% [10 of 17] head-of-bed elevation only vs. 77% [13 of 17] ranitidine only; RR 0.8, 95% CI 0.5 to 1.2).

Another crossover trial measured the change in GORD symptoms among 14 post-esophagectomy participants at 2 weeks on a 30-point score, the modified Dysfunction after Upper Gastrointestinal Surgery scale, with a higher score meaning a worse outcome [28]. It found a statistically significant improvement in GORD symptoms in the head-of-bed elevation group compared to the control group (mean difference in change scores − 5.1, 95% CI − 7.6 to − 2.6).

#### Intra-oesophageal pH measurement

We identified two trials evaluating the impact of head-of-bed elevation using ‘*bed blocks’* or ‘*sleeping on a wedge’* on the intra-oesophageal pH measurement (i.e. reported as the number of reflux episodes; percent of total time pH remained < 4; and acid clearance time) [25, 27].

A cross-over RCT of 15 participants [27], found a statistically significant reduction in acid exposure (defined as percent of total time intra-oesophageal pH remained ≤4) in both groups (mean % of total time: *‘sleeping on a wedge’* 15, 95% CI 3 to 26; *‘bed blocks’* 15, 95% CI 0 to 31; control 21, 95% CI 10 to 33); but not in the number of reflux episodes (defined as the total number of occasions of intra-oesophageal drop to < 4) and acid clearance time (defined as the total time that intra-oesophageal pH remained < 4 by the number of reflux episodes) in any of the two groups compared to the control group.

In addition to measuring GORD symptoms [25], participants in the ‘*bed blocks*’ arm in a cross-over study had statistically significant reductions in acid exposure (mean difference in % of total time that intra-oesophageal pH remained < 4, − 7.3, 95% CI − 13.9 to − 0.7) and reflux episodes (mean difference in number of drops in intra-oesophageal pH by two per participants, − 2.5, 95% CI − 4.2 to − 0.8) compared to participants in the control arm. But there were no statistically significant reductions neither in acid exposure (mean difference in % of total time, 1.2, 95% CI − 3.9 to 6.3) nor in reflux episodes (mean difference in number of episodes per participants, − 0.6, 95% CI − 2.3 to 1.1) among participants in the *‘sleeping on a wedge’* compared to the control.

#### Patient preferences

We identified two studies that measured patient preferences for head-of-bed elevation using ‘*bed blocks*’ or ‘*sleeping on a wedge’*. [27, 29] In a crossover trial [29], 36 of the 57 participants (63, 95% CI 51 to 76%) preferred the head-of-bed elevation to control intervention. Of the 15 participants included in another trial [27], 9 (60%) preferred *‘bed blocks’*; 5 (33%) preferred *‘sleeping on a wedge’;* and one (7%) preferred neither.

#### Adverse events

We identified two trials reporting adverse events associated with the intervention [26, 29]. A 2020 crossover RCT found that 39 of 63 participants (62%) in head-of-bed elevation group reported largely minor adverse events at 14 weeks compared to no one in the control group (24 bed-related e.g. bed slipping and unsteadiness; and 15 others e.g. varicose vain pain, musculoskeletal pain, and sexual activity interference) [29]. A factorial RCT found that two of 32 participants in the head-of-bed elevation group reported adverse events (one bed slipping and one sexual activity interference); both resolved and neither discontinued the intervention [26].

## Discussion

### Summary

We found five eligible controlled trials, evaluating the effect of head-of-bed elevation on GORD. Overall, the results suggest that head-of-bed elevation may have a beneficial effect on relieving gastroesophageal reflux symptoms. These results are supported by the observed improvement in physiological intra-oesophageal pH measurements. However, methodological and reporting issues limit the conclusions that can be drawn on the impact of head-of-bed elevation.

### Strengths and limitations

There are several limitations to our findings. The key limitation to our systematic review was the very small pool of existing evidence, and the poor quality and inadequate reporting of included trials. Most trials had small sample sizes and were of limited duration – none longer than 6 weeks – with considerable variations in the outcome measures, which precluded meta-analyses of the results from included studies. However, these are frequently reported challenges in trials evaluating non-drug interventions [30, 31]. The strengths of this review include its exhaustive search of multiple databases; inclusion of non-English language trials (i.e. minimising risk of language bias); rigorous quality assessment; and adherence to the Cochrane methodological standards [32] and PRISMA reporting guidance [33].

### Comparison with existing literature

A 2006 systematic review of lifestyles measures for gastroesophageal reflux [12] identified full reports from just two trials of head-of-bed elevation [25, 27] – both included in our review – which showed improvements in reflux symptoms and physiological intra-oesophageal pH measures. A 2016 systematic review published in Chinese, with a focus on patients experiencing reflux after treatment for oesophageal cancer, similarly concluded that head-of-bed elevation can improve reflux symptoms [34]. Two more recent studies from 2019 [28] and 2020 [29] were included in our systematic review.

### Implications for research and/or practice

The overuse of PPIs for gastroesophageal symptoms is a health problem, and a clinical challenge for clinicians globally. The findings of this review have confirmed that elevating the head of the bed is a cheap, easy-to-implement, relatively safe and promising approach. Importantly we did not find evidence of no benefit from this approach, but rather we found low quality evidence of benefit. Given the need to reduce the overuse of unnecessary medicines – with an unfavourable safety profile [35]– clinicians must be able to offer evidence-based non-pharmacological alternatives for treating these extremely common symptoms.

Additional quality randomized controlled trials evaluating the effect of head-of-bed elevation as part of a package of non-drug interventions in primary care settings are warranted. Future trials need to be of rigorous methodological quality with adequate sample size and duration to detect clinically meaningful differences and consistently measure and report patient-relevant outcomes. As de-prescribing and other initiatives targeting PPI overuse continue [1], offering clinicians and the public alternative non-drug approaches becomes critical.

## Conclusions

Methodological and reporting limitations in available literature preclude confident conclusions about the effect of head of bed elevation in relieving gastroesophageal symptoms. However, head of bed elevation could be still considered as a cheap, relatively safe, and promising alternative to drug interventions with unfavourable safety profiles.

## Supplementary Information

**Additional file 1: Appendix 1.** Search strategy and databases search string.

## Data Availability

The datasets used and/or analysed during the current study are available from the corresponding author on reasonable request.
