# Supplementary material for: Head of bed elevation to relieve gastroesophageal reflux symptoms: a systematic review
Source: BMC Fam Pract. 2021 Jan 19;22:24. doi: 10.1186/s12875-021-01369-0 (PMC7816499; doi:10.1186/s12875-021-01369-0)
Supplement: Supplementary file 1 — Additional file 1: Appendix 1. Search strategy and databases search string. [file 12875_2021_1369_MOESM1_ESM.docx]

**Title: Head of bed elevation to relieve gastroesophageal reflux symptoms: A systematic review**

*Loai Albarqouni^1^, Ray Moynihan PhD^2^, Justin Clark^3^,* Anna Mae Scott^4^*, Anne Duggan^5^, Chris Del Mar^6^*

# APPENDIX 1 – DATABASE SEARCH STRINGS

**Searches run 23/06/2020**

**PubMed (via the National Library of Medicine) search**

("Gastroesophageal Reflux"[Mesh] OR "Esophagitis"[Mesh] OR "Heartburn"[Mesh] OR "Dyspepsia"[Mesh] OR "Duodenogastric Reflux"[Mesh] OR Reflux[tiab] OR GERD[tiab] OR Oesophagitis[tiab] OR Esophagitis[tiab] OR Heartburn[tiab] OR “Heart burn”[tiab] OR GORD[tiab] OR Dyspepsia[tiab] OR Dyspepsias[tiab] OR Indigestion[tiab] OR Indigestions[tiab])

AND

("Sleep"[Mesh] OR Sleep[tiab] OR Sleeping[tiab] OR Bed[tiab] OR Bed-Head[tiab] OR Bed-up[tiab] OR Bedrest[tiab] OR Bed-rest[tiab] OR Nocturnal[tiab] OR Wedge[tiab])

AND

("Patient Positioning"[Mesh] OR "Supine Position"[Mesh] OR Positioning[tiab] OR Position[tiab] OR Supine[tiab] OR Elevation[tiab] OR Elevated OR Raised[tiab] OR Semi-recumbent[tiab] OR Semireclined[tiab] OR Semi-reclined[tiab] OR “Semi reclined”[tiab] OR Left-side[tiab] OR Right-side[tiab] OR Side[tiab] OR Lateral[tiab])

**Cochrane CENTRAL and trial registry (via Wiley) search**

([mh "Gastroesophageal Reflux"] OR [mh Esophagitis] OR [mh Heartburn] OR [mh Dyspepsia] OR [mh "Duodenogastric Reflux"] OR Reflux:ti,ab OR GERD:ti,ab OR Oesophagitis:ti,ab OR Esophagitis:ti,ab OR Heartburn:ti,ab OR "Heart burn":ti,ab OR GORD:ti,ab OR Dyspepsia:ti,ab OR Dyspepsias:ti,ab OR Indigestion:ti,ab OR Indigestions:ti,ab)

AND

([mh Sleep] OR Sleep:ti,ab OR Sleeping:ti,ab OR Bed:ti,ab OR BedHead:ti,ab OR Bedup:ti,ab OR Bedrest:ti,ab OR Nocturnal:ti,ab OR Wedge:ti,ab)

AND

([mh "Patient Positioning"] OR [mh "Supine Position"] OR Positioning:ti,ab OR Position:ti,ab OR Supine:ti,ab OR Elevation:ti,ab OR Elevated OR Raised:ti,ab OR Semirecumbent:ti,ab OR Semireclined:ti,ab OR "Semi reclined":ti,ab OR Side:ti,ab OR Lateral:ti,ab)

**Embase (via Elsevier) search**

('gastroesophageal reflux'/exp/mj OR 'esophagitis'/exp/mj OR 'Heartburn'/exp/mj OR 'Dyspepsia'/exp/mj OR 'Duodenogastric Reflux'/exp/mj OR Reflux:ti,ab OR GERD:ti,ab OR Oesophagitis:ti,ab OR Esophagitis:ti,ab OR Heartburn:ti,ab OR "Heart burn":ti,ab OR GORD:ti,ab OR Dyspepsia:ti,ab OR Dyspepsias:ti,ab OR Indigestion:ti,ab OR Indigestions:ti,ab)

AND

('Sleep'/exp/mj OR Sleep:ti,ab OR Sleeping:ti,ab OR Bed:ti,ab OR Bed-Head:ti,ab OR Bed-up:ti,ab OR Bedrest:ti,ab OR Bed-rest:ti,ab OR Nocturnal:ti,ab OR Wedge:ti,ab)

AND

('Patient Positioning'/exp/mj OR 'Supine Position'/exp/mj OR Positioning:ti,ab OR Position:ti,ab OR Supine:ti,ab OR Elevation:ti,ab OR Elevated OR Raised:ti,ab OR Semi-recumbent:ti,ab OR Semireclined:ti,ab OR Semi-reclined:ti,ab OR "Semi reclined":ti,ab OR Left-side:ti,ab OR Right-side:ti,ab OR Side:ti,ab OR Lateral:ti,ab)

AND

([embase]/lim)

**CINAHL (via EBSCO) search**

((MH "Gastroesophageal Reflux+") OR (MH "Esophagitis+") OR (MH "Heartburn+") OR (MH "Dyspepsia+") OR TI Reflux OR AB Reflux OR TI GERD OR AB GERD OR TI Oesophagitis OR AB Oesophagitis OR TI Esophagitis OR AB Esophagitis OR TI Heartburn OR AB Heartburn OR TI "Heart burn" OR AB "Heart burn" OR TI GORD OR AB GORD OR TI Dyspepsia OR AB Dyspepsia OR TI Dyspepsias OR AB Dyspepsias OR TI Indigestion OR AB Indigestion OR TI Indigestions OR AB Indigestions)

AND

((MH "Sleep+") OR TI Sleep OR AB Sleep OR TI Sleeping OR AB Sleeping OR TI Bed OR AB Bed OR TI Bed-Head OR AB Bed-Head OR TI Bed-up OR AB Bed-up OR TI Bedrest OR AB Bedrest OR TI Bed-rest OR AB Bed-rest OR TI Nocturnal OR AB Nocturnal OR TI Wedge OR AB Wedge)

AND

((MH "Patient Positioning+") OR (MH "Supine Position+") OR (MH "Lateral Position+") OR TI Positioning OR AB Positioning OR TI Position OR AB Position OR TI Supine OR AB Supine OR TI Elevation OR AB Elevation OR Elevated OR TI Raised OR AB Raised OR TI Semi-recumbent OR AB Semi-recumbent OR TI Semireclined OR AB Semireclined OR TI Semi-reclined OR AB Semi-reclined OR TI "Semi reclined" OR AB "Semi reclined" OR TI Left-side OR AB Left-side OR TI Right-side OR AB Right-side OR TI Side OR AB Side OR TI Lateral OR AB Lateral)
